# Correction: Wilderink et al. A Theoretical Perspective on Why Socioeconomic Health Inequalities Are Persistent: Building the Case for an Effective Approach. *Int. J. Environ. Res. Public Health* 2022, *19*, 8384

**DOI:** 10.3390/ijerph21050573

**Published:** 2024-04-30

**Authors:** Lisa Wilderink, Ingrid Bakker, Albertine J. Schuit, Jacob C. Seidell, Ioana A. Pop, Carry M. Renders

**Affiliations:** 1 Department of Health Sciences, Faculty of Sciences, Amsterdam Public Health Research Institute, Vrije Universiteit Amsterdam, 1081 HV Amsterdam, The Netherlands; j.c.seidell@vu.nl (J.C.S.); carry.renders@vu.nl (C.M.R.); 2 Department of Healthy Society, Windesheim University of Applied Sciences, 8017 CA Zwolle, The Netherlands; i.bakker@windesheim.nl; 3 School of Social and Behavioral Sciences, Tilburg University, 5037 AB Tilburg, The Netherlands; jantine.schuit@tilburguniversity.edu (A.J.S.); i.a.pop@tilburguniversity.edu (I.A.P.)

## Missing Citation

In the original publication [1], Oppong, S. Between Bandura and Giddens: Structuration theory in social psychological research? *Psychol. Thought* 2014, *7*, 111–123, was not cited. The citation has now been inserted in Section 3.2. Structuration Theory and should read as follows:

The theory from sociology proposes a bidirectional relationship between structure (environment) and agency (individual), where both can function as the cause and effect of the other (see Figure 6 [56]). Social structures create constraints and opportunities for particular behavior. At the same time, individuals are agents whose behavior creates and transforms social structures.

## Newly Added Reference

56. Oppong, S. Between Bandura and Giddens: Structuration theory in social psychological research? *Psychol. Thought* **2014**, *7*, 111–123.

Moreover, all subsequent reference citations were changed due to this inserted citation.

The authors apologize for any inconvenience caused and state that the scientific conclusions are unaffected. The original publication has also been updated.

## Figures and Tables

**Figure 6:**
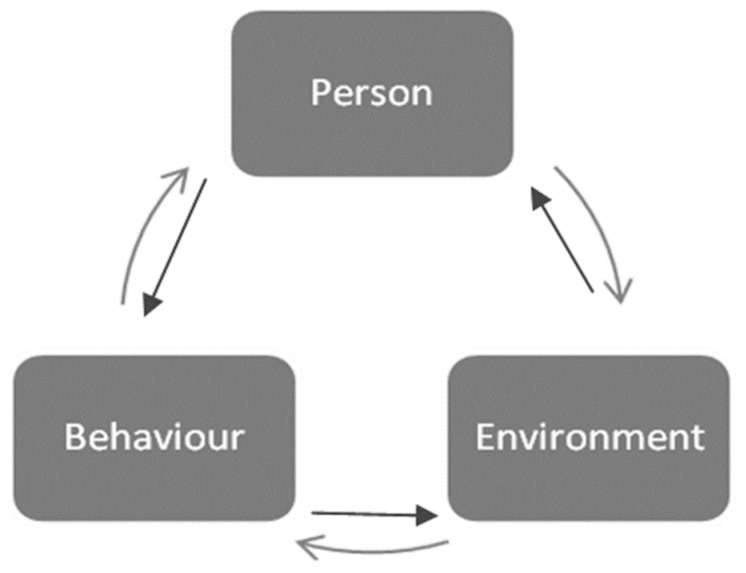
Structuration theory. Reproduced from Oppong, S., ‘Between Bandura and Giddens: Structuration Theory in Social Psychological Research?’; published by *Psychological Thought*, 2014 [56].
